# Novel Antiviral Agents: Synthesis, Molecular Modelling Studies and Biological Investigation

**DOI:** 10.3390/v15102042

**Published:** 2023-10-02

**Authors:** Simone Brogi

**Affiliations:** 1 Department of Pharmacy, University of Pisa, Via Bonanno 6, 56126 Pisa, Italy; simone.brogi@unipi.it; 2 Bioinformatics Research Center, School of Pharmacy and Pharmaceutical Sciences, Isfahan University of Medical Sciences, Isfahan 81746-73461, Iran

Representing more than 20% of all deaths occurring worldwide, infectious diseases remain one of the main factors in both human and animal morbidity and mortality. Approximately one-third of these deaths are attributable to viruses. Accordingly, over the past two decades, emerging and re-emerging viral agents, including the most recent SARS-CoV-2 and related coronaviruses (SARS and MERS), enteroviruses, Zika virus (ZIKV), and avian influenza A (H5N1, H1N1, and H7N9) viruses, have posed serious dangers to public health. Therefore, it is critical to identify novel antiviral drugs, vaccines, therapeutic strategies primarily based on the repurposing of marketed drugs, and early diagnostic and prevention strategies, considering the likelihood of future outbreaks. Recently, the World Health Organization (WHO) released a list of pathogens that could cause future pandemics and outbreaks. This list includes several viruses such as SARS-CoV-2 (COVID-19), Lassa fever, Ebola virus disease, Crimean-Congo hemorrhagic fever, Marburg virus disease, Middle East respiratory syndrome (MERS) and severe acute respiratory syndrome (SARS), ZIKV, Rift Valley fever, Nipah and henipaviral diseases, and Disease X. Furthermore, the WHO recommended that the scientific community invest in research to develop tests, treatments, and vaccines for the next outbreak [1].

In this case, computational techniques, such as cutting-edge machine learning methods, may speed up the development of efficient antivirals and therapeutic approaches. For this purpose, in July 2022, a Special Issue entitled “*Novel Antiviral Agents: Synthesis, Molecular Modelling Studies and Biological Investigation*” was launched, attracting the attention of researchers in the field. In this Special Issue, 15 papers on the development of antiviral agents against different types of clinically relevant viruses are published and in particular, 12 original research articles, 2 communication articles, and 1 review article are included. Accordingly, in this editorial article, we analyze and discuss the published papers, grouping them according to the research topic.

Starting from the studies conducted to identify possible antiviral agents against SARS-CoV-2 and related coronavirus species, Varbanov and coworkers synthesized and evaluated a series of antimicrobial diterenoids as possible anti-coronavirus agents. In particular, the authors synthesized a series of (+)-ferruginol derivatives using the abietane diterpene as a starting point because of its pharmacological profile, including its antimicrobial and, most importantly, antiviral activities. Compound C18-functionalized semisynthetic abietanes were synthesized from readily available methyl dehydroabietate and (+)-dehydroabietylamine. The resulting compounds were then computationally evaluated to assess the ADMET profile and select the most interesting derivatives for in vitro tests. Among the synthesized compounds, following the in silico indications, the authors selected eight compounds to evaluate their antiviral profile against human coronavirus 229E (HCoV-229E, PHE/NCPV-0310051v). The virus was established in the MRC-5 cell line, and its titers were determined as 50% infectious doses in the cell culture (CCID_50_/mL). Interestingly, two novel ferruginol analogs inhibited a cytopathic effect and significantly decreased viral titers. Notably, the bioactive compounds showed an interesting ADMET profile that encourages further research to evaluate possible activities in closely related virus families because they could have a broad range of antiviral effects [2]. Possible anti-SARS-CoV-2 agents were identified by Zhang and collaborators using artificial intelligence. In particular, to improve the performance of the transfer learning model for identifying SARS-CoV-2 Mpro inhibitors, the researchers adopted a data augmentation technique. On an external test set, this approach, based on a deep learning technique, appeared to perform better than Chemprop, random forest, and graph convolutional neural network. Using the developed computer-based model, naturally and de novo produced compound libraries were screened. As screening results, by combining this method with other in silico analyses (e.g., molecular docking and PAINS evaluation), twenty-seven molecules were submitted to biological evaluation to confirm anti-Mpro activity. Gossypol acetic acid and hyperoside, two of the selected computational hit compounds, both exhibited inhibitory activity against SARS-CoV-2 Mpro (gossypol acetic acid IC_50_ = 67.6 μM; hyperoside IC_50_ = 235.8 μM). Despite having micromolar potency, these two compounds provide useful scaffolds for future drug optimization in the fight against COVID-19. Accordingly, the findings of this study may offer a useful method for identifying lead compounds to develop anti-SARS-CoV-2 and other coronavirus agents. Remarkably, artificial intelligence in drug discovery is increasingly being used, considering the significant advantages in lowering the costs and time associated with drug research and the development trajectory, particularly for emerging diseases [3]. Hassan and colleagues used a combined approach that coupled computer-based and experimental methods to identify potential TMPRSS2 inhibitors. This approach is very interesting because, considering that host proteases are necessary for coronaviruses to facilitate the entry of the virus into host cells, targeting the conserved host-based entry process, as opposed to the viral proteins that are constantly changing, may have advantages. Camostat and nafamostat have been identified as TMPRSS2 inhibitors, showing irreversible binding to the target protein. Considering the chemical structure of nafamostat, the researchers rationally designed a small series of related rigid analogs that were evaluated in silico using a molecular docking study employing the experimentally solved structure of TMPRSS2 (PDB: 7MEQ). The computer-based evaluation provided the compounds to be prioritized for synthesis. Accordingly, six of the best predicted molecules were synthesized using pentamidine as a starting point and experimentally validated in vitro. Four of them showed some bioactivities. Two compounds effectively inhibited TMPRSS2 in the micromolar range, but they showed modest activity in cell-based assays. Interestingly, although one compound did not show appreciable inhibitory activity against TMPRSS2, it exhibited possible cellular activity in a low micromolar range (IC_50_ = 10.87 µM) in inhibiting the fusion of membranes, indicating that another molecular target may be responsible for its effect. Overall, the results of this investigation provided hit compounds that might be used as a basis for the discovery of novel viral entry inhibitors with potential usage against coronaviruses [4]. Mohammad and colleagues developed a virtual screening protocol for identifying possible antiviral agents targeting SARS-CoV-2 NSP3 macrodomain-1, which plays a crucial role in viruses’ attack on the innate immune system. The authors virtually screened, against the mentioned target, a library of natural compounds using different computer-based techniques such as molecular dynamic (MD) simulations (100 ns) coupled to the MM/GBSA calculation for estimating binding free energies. Among the screened compounds, the authors identified two promising hit compounds (3,5,7,4′-tetrahydroxyflavanone 3′-(4-hydroxybenzoic acid) and 2-hydroxy-3-O-beta-glucopyranosyl-benzoic acid), showing a stable binding mode within the SARS-CoV-2 NSP3 macrodomain-1 binding site, with significant binding affinity (molecule A ΔG_bind_ = −61.98 ± 0.9 kcal/mol; molecule B ΔG_bind_ = −45.125 ± 2.8 kcal/mol). To confirm the predicted activity of the selected drugs, computational bioactivity evaluation and dissociation constant (K_D_) measurements were performed for both complexes. The findings highlighted that these two potential antivirals could block SARS-CoV-2 NSP3 macrodomain-1 functions, thereby directly boosting the host’s immune response [5]. Finally, in an article featuring communications, Nydegger and coworkers described the application of microscale thermophoresis (MST) as a reliable binding assay to be employed in coronavirus drug discovery. This article is important because considering that new SARS-CoV-2 mutations keep surfacing as the COVID-19 epidemic spreads, the necessity is emerging of creating efficient methods to research such variants, as well as the emergence of future potential coronaviruses. This approach could be helpful in the development of novel antivirals. The researchers applied MST in three different types of tests: (a) binding of the SARS-CoV-2 spike receptor binding domain (RBD) to the host target ACE2; (b) binding of RBD to ACE2 in complex with the amino acid transporter SLC6A20/SIT1 and the mutated rs61731475 (I529V); and (c) binding of peptide-based agents to RBD as an approach to preclude virus entry. The findings showed that MST is an extremely accurate method for investigating protein–ligand and/or protein–protein interactions in anti-coronavirus drug discovery, indicating that it is a perfect technique for examining viral variations to develop effective antivirals. Furthermore, the MST approach can be used to examine whether or not membrane proteins are expressed in intracellular membranes. Notably, the strategy outlined in this study could be applied to different coronaviruses, including novel viral variants [6].

Dengue virus (DENV), which has a high global incidence of infections, causes dengue, an acute febrile illness. No medication is currently available for the treatment of this disease. Accordingly, Garcia-Ariza and coworkers reported a virtual screening protocol for identifying indole-containing molecules from natural compounds able to inhibit the DENV NS5 protein, evaluating its antiviral potential in vitro. To this end, using AutoDock Vina software, molecular docking on NS5 was performed, and chemical agents with interesting ADMET properties and pharmacological profile, assessed using the SwissADME web server, were chosen. NS1 protein expression was measured to assess its preliminary antiviral activity. Furthermore, NS5 production, utilizing the DENV-2 Huh-7 replicon using ELISA, and the quantification of viral RNA, using RT-qPCR, were used to assess the impact on viral genome replication and/or translation. The results of virtual screening identified 15 possible DENV NS5 inhibitors. Among them, the computational hit M78 exhibited a strong effect on the lifecycle of the DENV-2 virus. Compound M78 tested at a concentration of 50 µM reduced viral RNA (1.7 times), whereas NS5 protein expression was reduced by 70%, indicating that the replication and/or translation of the viral genome is strongly influenced by M78. Accordingly, studies of M78 function in NS5, pre-treatment activity, and virucidal effects on additional Dengue serotypes and flaviviruses are highly desired [7].

Castro-Amarante and colleagues explored the potential anti-ZIKV effects of the C-terminal region of the E protein (stem) from DENV. Notably, among flaviviruses, this region is highly conserved; consequently, it is attractive for developing peptide-based antiviral agents. Considering that the stem region of the ZIKV and DENV viruses share this sequence, the purpose of the research was to assess the cross-inhibition of ZIKV by the stem-based DV2 peptide (419–447), which has previously been demonstrated to inhibit DENV serotypes. Because the stem regions of the DENV and ZIKV viruses are similar, the researchers investigated whether the stem-based DV2 peptide (419–447), which has been demonstrated to inhibit all DENV serotypes, may also inhibit ZIKV. To determine the antiviral effect of the DV2 peptide against ZIKV, the researchers conducted in vitro and in vivo experiments. Computer-based methods were used to explore the binding mode of the DV2 peptide. The findings clearly indicated that the DV2 peptide was able to target amino acid residues exposed on the surface of the ZIKV envelope (E) protein. The DV2 peptide effectively reduced ZIKV infectivity in the Vero cell line, but had no discernible harmful effects on eukaryotic cells. Notably, the DV2 peptide also decreased mortality and morbidity in mice severely infected with a ZIKV strain discovered in Brazil. Overall, the findings of this work indicated that the DV2 peptide could be a promising agent for treating ZIKV infections, possibly preventing or reducing neurological impairment in neonates linked to infection in pregnant women. Moreover, this work provided indications for developing anti-flavivirus drugs from synthetic stem-based peptides [8].

Scior and collaborators focused their work on the discovery of possible antiviral agents against the human influenza A virus. The researchers developed a computer-based protocol to virtually screen a commercial chemical library containing 660,961 chemical entities against the drug target of human influenza A virus, neuroaminidase (viral strain A/Vietnam/1203/2004; H5N1). By applying a series of sequential filters, such as molecular fingerprints, pharmacophore modeling, molecular docking, and ADMET profile prediction, virtual screening was performed, taking into account several reference compounds for neuroaminidase (natural substrate sialic acid, substrate-like DANA, known antivirals such as peramivir, laninamivir, zanamivir, and oseltamivir). Notably, prior to use, all computational filtering tools underwent validation. Two of the top compounds currently have successful patent filings. Accordingly, the identified lead compounds could be used for further optimization to develop more focused ligands that act as antivirals against the human influenza A virus [9].

Next, two works published within this Special Issue involved the human immunodeficiency virus (HIV-1) to identify possible anti-HIV agents. In particular, Alvarez-Rivera and coworkers described the characterization of a bioactive extract polysaccharide peptide (PSP) from *Coriolus versicolor* by quantitative proteomic analysis. In THP1 monocytic cells, PSP showed anti-HIV replicative capabilities, although the antiviral mechanism were not elucidated. Accordingly, the researchers explored the possible mechanisms by which PSP exerts an anti-HIV profile. In this particular article, the authors investigated the involvement of PKR and IRE1α in the phosphorylation of cofilin-1 and their HIV-1-restriction functions in THP1. The infected supernatant was used to evaluate the HIV-1 p24 antigen to assess PSP’s potential for restriction. Immunoblots were used to test the biomarkers PKR, IRE1α, and cofilin-1, and proteome biomarkers were validated using RT-qPCR. Furthermore, Western blot analysis was performed to confirm viral entrance and cofilin-1 phosphorylation using PKR/IRE1 inhibitors. The results demonstrated that treatment with the bioactive peptide PSP reduced viral infectivity, indicating that cofilin-1 phosphorylation and viral limitation were controlled by PKR and IRE1, which is reflected in the early entrance phase. Accordingly, new molecular insights into how UPR, IFN-IP, and cytoskeletal events interact are provided by the current study. Interestingly, the information presented in the article suggested that PSP may be utilized as a natural alternative to stop HIV-1 entrance [10]. In a communication article authored by Zhu and collaborators, the mechanism of the molecular recognition of the HIV-1 gp120 V3 loop by the coreceptor CXC chemokine receptor 4 (CXCR4) was investigated using a construct containing the V3 loop (full-length). It has been established that when HIV-1 enters a cell, one of its main coreceptors, CXCR4, is recognized by the virus through the third variable loop (V3 loop) of the HIV-1 envelope glycoprotein, gp120. In this study, a disulfide bond was used to covalently connect two ends of the V3 loop, creating a constrained cyclic peptide. Furthermore, an all-D-amino acid analog of the L-V3 loop peptide was created to test the impact of the altered conformation of the side-chains of the peptide on CXCR4 recognition. Similar binding recognition was shown for both of these cyclic L- and D-V3 loop peptides to the CXCR4 receptor, but not to the CCR5 receptor, indicating their selective interactions with CXCR4. Computational studies have highlighted that numerous negatively charged Asp and Glu residues on CXCR4 play crucial roles, likely through electrostatic interactions with the positively charged Arg residues found in these peptides. These findings confirm the flexibility of the HIV-1 gp120 V3 loop-CXCR4 interface for ligands of various chiralities, which may be important for the virus’s capacity to maintain coreceptor recognition despite mutations at the V3 loop. The discovery of D-amino acid accommodation by the CXCR4 ligand-binding surface paves the way for the creation of more stable D-peptide analogs for antiviral use [11].

The foot-and-mouth disease virus (FMDV), a (+)-ssRNA virus that belongs to the Picornaviridae family, represents a commercially significant pathogen that affects livestock with cloven hooves. Theerawatanasirikul and coworkers utilized a computer-based approach to identify possible inhibitors of FMDV RNA-dependent RNA polymerase (RdRp) (3Dpol). The authors evaluated 5596 compounds in silico using a blind docking approach against 3Dpol, and selected 21 compounds with satisfactory predicted binding affinity. The selected computational hits were tested in vitro, and four compounds (NSC65850, NSC670283 (spiro compound), NSC217697 (quinoline), and NSC292567 (nigericin)) exhibited dose-dependent antiviral effects in vitro (BHK-21 cell-based test), showing an EC_50_ ranging from 0.78 to 3.49 µM. Notably, without affecting FMDV’s primary protease, 3Cpro, these substances were able to strongly inhibit FMDV 3Dpol activity in the cell-based 3Dpol inhibition experiment with significant IC_50_ values ranging from 0.8 nM to 0.22 µM. The drugs’ 3Dpol inhibitory activities were dose dependently commensurate with the reduced viral load and generation of (−)-ssRNA. Finally, the authors have discovered possible FMDV 3Dpol inhibitors that bind to the active areas of the enzyme and could prevent viral multiplication. These substances may be helpful in the treatment of FMDV and other picornaviruses [12].

The lack of therapeutic options for clinical isolates of carbapenem-resistant *Pseudomonas aeruginosa* (CRPA) necessitates the development of novel therapeutic approaches. Accordingly, Mabrouk and colleagues characterized and evaluated two locally isolated phages in a hydrogel formulation in vitro and in vivo and directed them against CRPA clinical isolates. Both phages were examined using phenotypic, genomic, in vitro, and rat models of cutaneous thermal damage caused by *Pseudomonas aeruginosa*. The two siphoviruses, vB_Pae_SMP1 and vB_Pae_SMP5, are members of the Caudovirectes class. Each phage showed an icosahedral head measuring 60 ± 5 nm and a flexible, non-contractile tail measuring 170 ± 5 nm, with the exception of vB_Pae_SMP5, which had an extra base plate with a 35 nm fiber visible at the tail’s end. The phage lysate from CRPA propagation was combined with 5% *w*/*v* carboxymethylcellulose (CMC), which has a spreadability coefficient of 25 and a titer of 108 PFU/mL, to prepare the hydrogel. In the groups treated with Phage vB_Pae_SMP1, vB_Pae_SMP5, or a two-phage cocktail, hydrogel cellular subepidermal granulation tissues with remarkable records of fibroblastic activity and mixed inflammatory cell infiltrates were present. These tissues also showed records of 17.2%, 25.8%, and 22.2% dermal mature collagen fibers. Overall, phage vB_Pae_SMP1 or vB_Pae_SMP5, or the two-phage cocktails made into hydrogels, were effective at controlling CRPA infection in burn wounds and promoting healing at the injury site, as shown by histological inspection and a reduction in animal death rate. Thus, each phage lysate or the two-phage cocktail created as a hydrogel is a useful topical recipe to utilize in burn wounds after severe CRPA infections. To ensure that the corresponding phage hydrogels are appropriate for clinical use in humans, more clinical investigations should be conducted [13].

Fabrizi and colleagues described the possible use of pan-genotypic antivirals to treat hepatitis C virus (HCV) in chronic kidney disease. It is well-established that patients with chronic renal disease (CKD) often contract HCV. Recent research has shown that adult populations with chronic HCV are at increased risk of developing CKD. A relationship between positive anti-HCV serologic status and an elevated incidence of CKD was shown by combining the findings of longitudinal research (n = 2,299,134 distinct individuals) in a systematic review with a meta-analysis of clinical investigations (overall adjusted HR estimated was 1.54 (95% CI, 1.26–1.87), *p* = 0.0001). Furthermore, according to new guidelines, pan-genotypic medications, which are effective against all HCV genotypes, should be used as the initial HCV therapy because they have the potential to be both efficient and secure even in the presence of CKD. A paradigm shift in the treatment of HCV infection has been brought about by the development of direct-acting antiviral medications. The objective of the selected report was to present the most pertinent information regarding pan-genotypic direct-acting antiviral drugs in advanced CKD (CKD stages 4 or 5). Accordingly, through computerized databases and the gray literature, the authors gathered studies using several keywords (e.g., ‘Hepatitis C’ AND ‘Chronic kidney disease’ AND ‘Pan-genotypic agents’). The results of this investigation showed that glecaprevir/pibrentasvir (GLE/PIB) and sofosbuvir/velpatasvir (SOF/VEL) are the two most significant pan-genotypic drug combinations for treating HCV in severe CKD. GLE/PIB combined therapy in CKD stage 4/5 provided SVR12 rates ranging from 86% to 99%, according to two clinical trials (EXPEDITION-4 and EXPEDITION-5) and other “real-world” studies (n = 6). Clinical trials (n = 1) and “real life” studies (n = 6) demonstrating the effectiveness of SOF/VEL were retrieved. The results showed that the SVR rate in experiments using the SOF/VEL antiviral combination was 100%. In patients on SOF/VEL, the dropout rate (caused by AEs) ranged from 0% to 4.8%. Although there are currently few trials on this topic with small sample sizes, pan-genotypic drug combinations, such as SOF/VEL and GLE/PIB, seem efficient and secure for treating HCV in progressed CKD. Studies are being conducted to determine if effective antiviral treatment with direct-acting antiviral drugs will result in improved survival in individuals with advanced CKD [14].

The WHO global eradication initiatives are aimed at eliminating poliovirus (PV), the cause of poliomyelitis. Following the elimination of type 2 and 3 wild-type PVs, vaccine-derived PV and type 1 wild-type PV, remain as an important threat to eradication. Therefore, antivirals could be used to effectively control a possible outbreak; nonetheless, no anti-PV medicines are available on the market. In this context, Arita and collaborators developed a virtual screening of a chemical library of compounds present in edible plant extracts (6032 extracts) to identify anti-PV agents. The results showed that anti-PV action was discovered in extracts from seven distinct plant species. Chrysophanol and vanicoside B (VCB) extracted from *Rheum rhaponticum* and *Fallopia sachalinensis* were identified as promising anti-PV agents. VCB inhibited the activity of PI4KB in vitro (IC_50_ = 5.0 μM) while targeted the host PI4KB/OSBP pathway for anti-PV action (EC_50_ = 9.2 μM). Furthermore, additional studies are expected to confirm the antiviral potential of VCB. Although the anti-PV activity was clearly specific to the PI4KB/OSBP pathway, we could not rule out the possibility that the observed anti-PV activity was influenced by the off-target effect of VCB (CC_50_ = 27 μM) that is reflected in a limited therapeutic window (total protection from infection caused by PV1[Sabin 1] at 20 μM) [15].

Finally, in the last studies to be included in this Editorial article, Manna and collaborators presented a review article that explored the antiviral profile of various oxa- and aza-heterocycles. The main goal of this review article was to explore the biological importance, synthetic pathways, and antiviral activity of these ring structures. Furthermore, the structure–activity relationship (SAR) of the selected molecules is presented, together with a list of their key features, to create a useful framework for scientists in the field. The synergistic findings are crucial for introducing innovative instruments for the next antiviral drug discovery programs [16].

The *Viruses* Editorial staff, all the authors and co-authors for their significant contributions to this Special Issue, and all the reviewers for their work in evaluating the submissions deserve my sincere gratitude as Guest Editor. The success of the research topic as a whole was a result of all of these combined efforts. We hope that this subject will assist scientists in the development of efficient antivirals and serve as a substantial source of knowledge and inspiration for researchers and students. You may obtain this Special Issue for free by visiting the following link: https://www.mdpi.com/journal/viruses/special_issues/novelantiviral_agents.
